# Enhanced Erbium-Doped Ceria Nanostructure Coating to Improve Solar Cell Performance

**DOI:** 10.3390/ma8115399

**Published:** 2015-11-12

**Authors:** Nader Shehata, Michael Clavel, Kathleen Meehan, Effat Samir, Soha Gaballah, Mohammed Salah

**Affiliations:** 1 Bradley Department of Electrical and Computer Engineering, Virginia Polytechnic Institute State University, 302 Whittemore Hall, VA 24061, USA; mbclavel@vt.edu; 2 Department of Engineering Mathematics and Physics, Faculty of Engineering, Alexandria University, Elhadara, Alexandria 21544, Egypt; m.salah@mena.vt.edu; 3 Center of Smart Nanotechnology and Photonics (CSNP), Smart Critical Infrastructure (SmartCI) Research Center, Alexandria University, Elhadara, Alexandria 21544, Egypt; effat_samir@mena.vt.edu (E.S.); eng.soha.gaballah@gmail.com (S.G.); 4 School of Engineering, University of Glasgow, Glasgow, Scotland G12 8QQ, UK; Kathleen.Meehan@glasgow.ac.uk; 5 Department of Electrical Engineering, Faculty of Engineering, Alexandria University, Elhadara, Alexandria 21544, Egypt; 6 Department of Chemical Engineering, Faculty of Engineering, Alexandria University, Elhadara, Alexandria 21544, Egypt

**Keywords:** ceria nanoparticles, erbium dopant, up-conversion, conductivity, solar cells

## Abstract

This paper discusses the effect of adding reduced erbium-doped ceria nanoparticles (REDC NPs) as a coating on silicon solar cells. Reduced ceria nanoparticles doped with erbium have the advantages of both improving conductivity and optical conversion of solar cells. Oxygen vacancies in ceria nanoparticles reduce Ce^4+^ to Ce^3+^ which follow the rule of improving conductivity of solar cells through the hopping mechanism. The existence of Ce^3+^ helps in the down-conversion from 430 nm excitation to 530 nm emission. The erbium dopant forms energy levels inside the low-phonon ceria host to up-convert the 780 nm excitations into green and red emissions. When coating reduced erbium-doped ceria nanoparticles on the back side of a solar cell, a promising improvement in the solar cell efficiency has been observed from 15% to 16.5% due to the mutual impact of improved electric conductivity and multi-optical conversions. Finally, the impact of the added coater on the electric field distribution inside the solar cell has been studied.

## 1. Introduction

Optical nanostructures that emit visible light when excited by ultra-violet (UV) or infrared (IR) photons have been extensively studied for solar energy applications [1,2]. Recent research on one of these nanomaterials, cerium oxide (ceria) nanoparticles, has shown that its material properties are extremely well suited for a lot of applications [3,4,5,6]. Visible emission from either UV excitation (down-conversion) or IR excitation (up-conversion) can be obtained from ceria nanoparticles. However, both up- and down-conversion processes involve different physiochemical properties in ceria and optimization of each optical process via various nanoparticle synthesis and post-growth procedures tends to quench the efficiency of the other process.

Coating solar cells or panels with nanostructures has been recently investigated to enhance the conversion efficiency of the cells [7,8]. In this paper, it is aimed to coat a polycrystalline silicon cell with a thin layer of reduced erbium-doped ceria nanoparticles to improve the cell efficiency. The synthesized reduced erbium-doped ceria nanoparticles (REDC NPs) would have two main characteristics: to have higher conductivity and to be applicable for optical up- and down-conversions. In detail, the synthesized doped ceria nanoparticles would have relatively high concentrations of tri-valent cerium ions in trap states with a higher concentration of oxygen vacancies. When coated on the solar cells, the synthesized reduced ceria could have higher conductivity and improve the mobility of the generated photoelectrons, due to the increased rate of cerium ion conversion from +4 to +3 states accompanied with an increasing creation rate of charged O-vacancies. In addition, the reduced erbium-doped ceria nanoparticles have the unique material properties to act as an optical medium for both down-conversion and up-conversion at the same time to generate multi-wavelength visible emissions under near-UV and IR excitations, respectively. “Reduced” means that the nanoparticles are synthesized under a reduction environment using hydrogen. This environment helps to form oxygen vacancies and cerium ions (+3 states). These cerium tri-valent trap states are responsible for optical down-conversion. However, “non-reduced” means that there is no hydrogen during synthesis, which would not form the Ce^3+^ states. Then, without the reduction environment, the erbium-doped ceria nanoparticles are abbreviated EDC NPs. Then, the used synthesis process results in a high concentration of Ce^3+^ ions associated with the oxygen vacancies in ceria, which is required to obtain high fluorescence efficiency in the down-conversion process. Simultaneously, the synthesized nanoparticles contain the molecular energy levels of erbium that are required for up-conversion. Therefore, REDC NPs which are synthesized using this procedure can emit visible light when excited with either or both UV or IR photons. The synthesized nanoparticles were analyzed using optical absorbance spectroscopy, direct band gap calculations, fluorescence spectroscopy, transmission electron microscope (TEM), X-ray diffraction (XRD) and the electrical conductivity measurement. Then, the synthesized reduced nanoparticles were coated on polycrystalline silicon cells for improving the cell efficiency, which has been proved through I–V analysis, in addition to other cell characteristics such as open circuit voltage, short circuit current, and fill factor. Also, rate generation and E-field distributions of the coated cell were analyzed. Compared to other ceria nanostructure coatings in the literature [9,10,11], our novel coating offers the simultaneous enhancement of both optical and conductive properties which leads to improving the solar cell's efficiency without considering the traditional anti-reflection coatings. In addition, our synthesized nanoparticles are relatively low-cost and easy to prepare with a simple chemical synthesis procedure with a simple coating technique.

## 2. Results and Discussion

### 2.1. Nanoparticles Characterization

**Figure 1:**
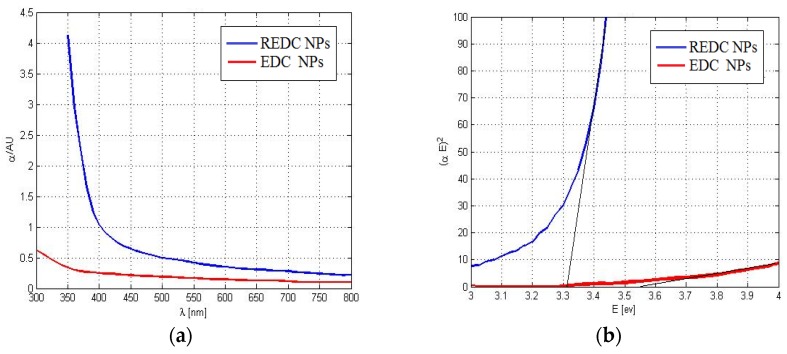
(**a**) Absorbance dispersion curves for reduced nanoparticles (REDC NPs) annealed at 700 °C and the non-reduced nanoparticles (EDC NPs); (**b**) the corresponding direct band gap of both REDC NPs and EDC NPs.

**Figure 2:**
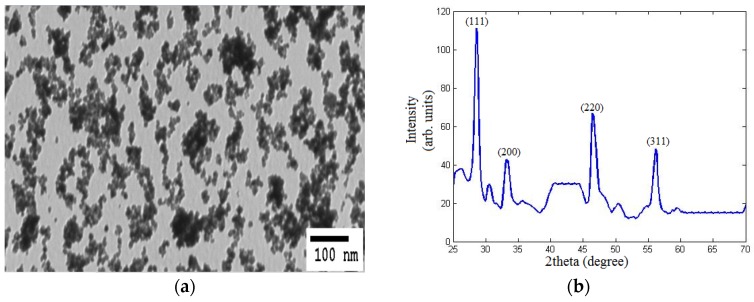
(**a**) TEM image and (**b**) XRD pattern of REDC NPs at annealing temperature of 700 °C.

The optical absorption spectra of the synthesized REDC NPs are plotted in Figure 1a. Consequently, the corresponding values for the calculated allowed direct band gaps of the annealed samples are shown in Figure 1b, using Equation (1) [12,13].

(1) $$\propto E=A{\left(E-E_{g}\right)}^{1/2}$$

where α is the absorbance coefficient, *A* is a constant dependent on the effective masses of electrons and holes of the material, *E* is the absorbed photon energy, and *E_g_* is the direct allowed band gap. Thus, from the absorbance dispersion results in Figure 1a, (∝E)^2^ is presented *versus* the photon energy (*E*) as shown in Figure 1b. Then, the intersection between the extension of the linear region of the resulted curve with the *E*-axis indicates the band gap. Compared to the non-reduced nanoparticles, it can be observed that the band gap of the annealed nanoparticles is biased towards 3 eV, which is approximately the band gap energy for Ce_2_O_3_. Thus, there is evidence for the formation of a higher concentration of Ce^3+^ with corresponding oxygen vacancies [14]. The annealed REDC NPs are imaged using TEM as shown in Figure 2a. The mean diameter is found to be 10 nm, which shows that our synthesized REDC NPs are smaller than other optical nanoparticles that have been studied as an optical active medium for down- or up-conversion [15,16], which can lead to both better conductive and optical properties due to a higher surface-to-volume ratio. The XRD pattern is presented in Figure 2b, measured on a sample of the REDC NPs annealed at 700 °C, to demonstrate that the predominant nanostructure of the REDC NPs is cerium dioxide [17,18]. From the measurement of the width of individual intensity peaks, the average size (*t*_XRD_) of nanoparticles, or the diameter in case of spherical-shaped nanoparticles, can be calculated from Scherrer’s equation [13]

(2) $$t_{\text{XRD}}=\frac{0.9\text{λ}}{\text{β}cos\text{θ}}$$

where λ is the wavelength of the incident X-rays (0.15406 nm), β is the full-width half-maximum (FWHM), and θ is the diffraction angle. From the first peak, which represents the most stable plane of ceria (111), the average size of the nanoparticles is found to be ~10 nm.

**Figure 3:**
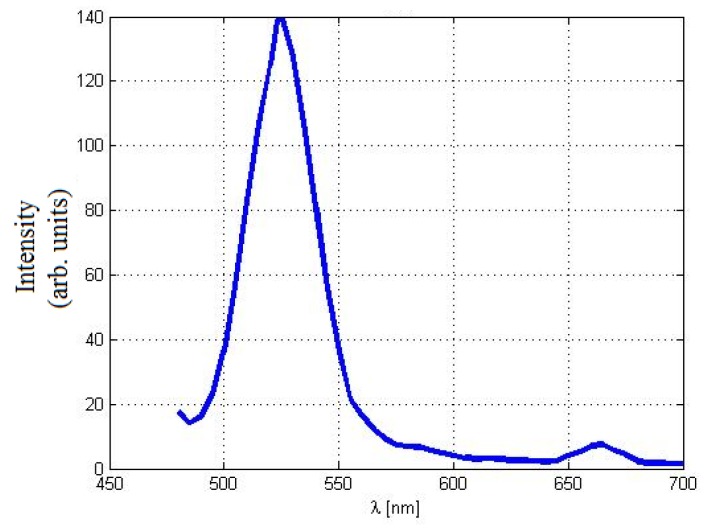
Emission spectrum of REDC NPs under simultaneous excitations of both near-UV (430 nm) and IR (780 nm) excitations.

Under the simultaneous emission of both near-UV (λ = 430 nm) and IR (780 nm) excitations, the dominant visible emission from the EDC NPs is centered around 520 nm with a relatively smaller-peak emission at 670 nm, as shown in Figure 3. This emission peak is including both contributions; the down-conversion one which involves the radiative relaxation of the 5d–4f transition of an excited Ce^3+^ ion in Ce_2_O_3_ resulting in the broadband emission of the green wavelength [19]. As the synthesized REDC NPs that contain some fraction of Ce_2_O_3_ are illuminated with near-UV light, then some fraction of the valence band electrons are excited to an oxygen vacancy defect state located within the CeO_2_ band gap. Regarding the second contribution of the up-conversion process, erbium ions form stable complexes with oxygen in the ceria host during the annealing, and the crystalline structure of the nanoparticle improves, both of which increase the efficiency of Er^3+^ ions to behave as optically active centers for up-conversion emissions with the mutual contribution of green light in addition to the low red emission [15,19]. In addition, the conductivity of the REDC NPs is measured to be 77 μS/cm, which is found experimentally to be ~22% higher than the conductivity of normal EDC NPs, 63 μS/cm.

**Figure 4:**
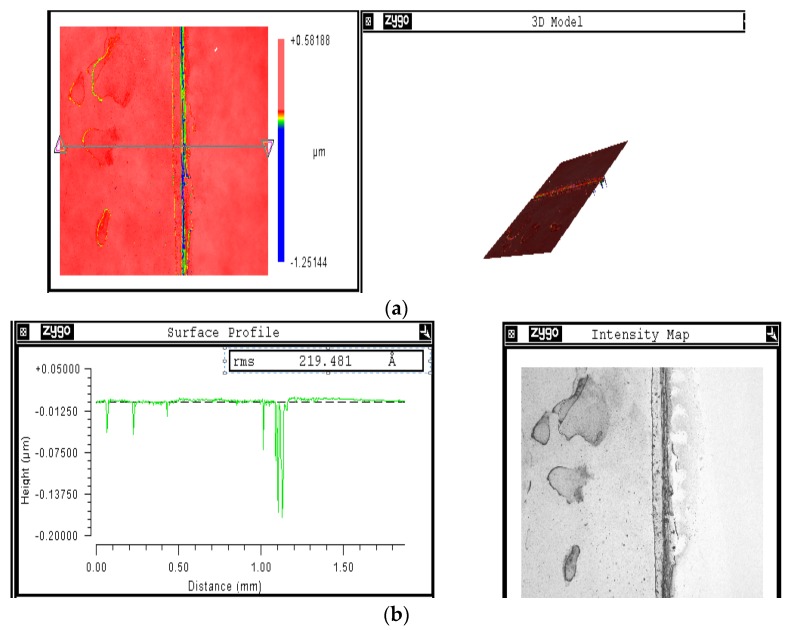
(**a**) Surface profile of coated cell at the edge between coating and the electrode and (**b**) the profile distribution with the intensity map.

The surface profile of the coated cell is shown in Figure 4, with focus on the region between the electrode and the coated edge. It could be observed that the mean thickness of the coating is around 20 nm with quite a non-uniform distribution of the coating, as shown in Figure 4b regarding the intensity imaging, which may be due to the spin coating technique itself. This coating technique could be considered as a trade-off between surface uniformity and simplicity. However, other coating techniques may lead us to miss the conductivity of the nanostructures due to missing oxygen vacancies with the conversion of Ce^3+^ to Ce^+4^.

### 2.2. Coated Solar Cell Characterization

**Figure 5:**
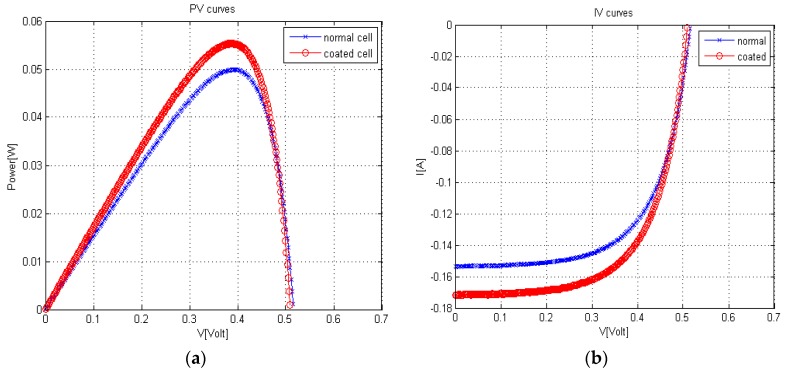
(**a**) P–V curve and (**b**) I–V curve of silicon solar cells in both the uncoated (normal) case and those coated with REDC NPs.

As investigated in the previous sections, coating the back side of a silicon solar cell with REDC NPs has the advantages of improving multi-optical conversions, leading to the conversion of some UV and IR wavelengths that solar cells cannot absorb to visible light wavelengths which can be absorbed. Figure 5a,b show the improvement in P–V and I–V curves, respectively, after coating the cell with REDC NPs. The promising comparison between coated and uncoated cells was shown in Table 1, and it clearly shows that power conversion efficiency (PEC) has been improved from 15.1% to 16.7%, which is about a 10.8% improvement of cell efficiency due to coating compared to uncoated cells. As can be noticed from Table 1, there is a relatively high improvement of short circuit current (*I_s.c_*) with the effect of our synthesized nanoparticle coating compared to both quite stable open circuit voltage (*V_o.c_*) and fill factor (*F.F*). Overall, the increase in the current, and consequently the power, could be explained due to the increase in the rate of photoelectrons, whether through a higher generation rate due to optical conversions and/or the better mobility due to a conductive nanostructure coating.

**Table 1:** Comparison between coated and un-coated cells.

Condition	$V_{o.c}$	$I_{s.c}$	*F.F*	η %
Uncoated	0.5155	0.1537	0.6301	15.1075
coated cell	0.5095	0.1718	0.6322	16.7452

**Figure 6:**
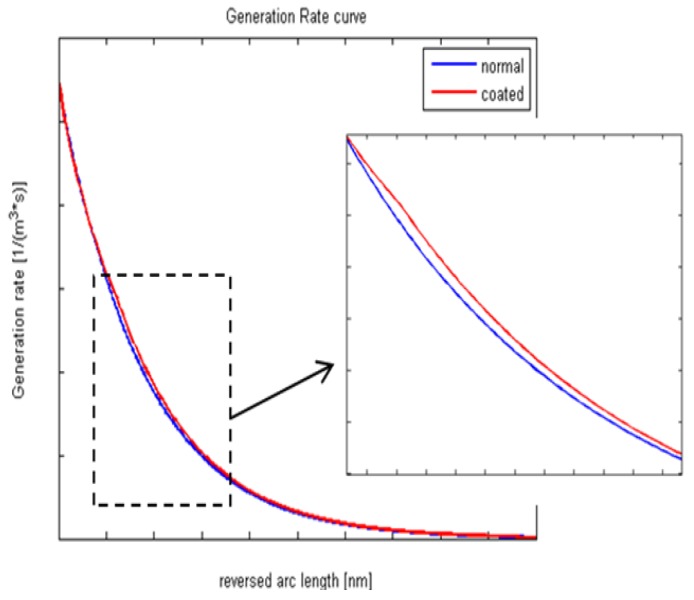
Normalized generation rate of silicon cells with/without REDC NP coating layer.

**Figure 7:**
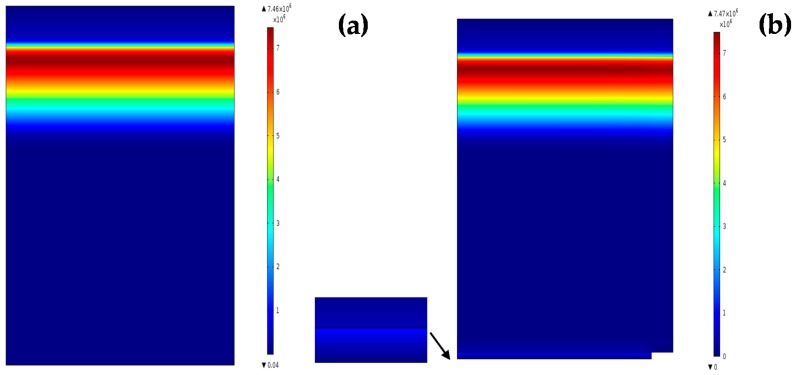
Electric field distribution (**a**) before and (**b**) after REDC NP coating.

Beside the advantage of multi-optical conversions of REDC NPs, these nanoparticles have the ability to improve the electrical conductivity of the generated photoelectrons of solar cells through the great number of formed O-vacancies. Then, we aimed to simulate Si solar cells before and after the REDC NP layer coating through studying the normalized generation rate and field distribution. Figure 6 shows the difference in generation rate curves, and the surface electric field distributions are shown in Figure 7a,b. A simulation model has been built in a two-dimensional (2D), semiconductor module. This model deals with REDC NPs as it is a conductive layer with a band gap $E_{g}\;=\;3.31\;$eV, room temperature conductivity $\text{σ}=77\;\times 10^{-6}\text{ S}/\text{cm}$, and electron mobility ${\text{μ}}_{\text{e}}=2.8\;\times \;10^{-7}{\text{ cm}}^{2}/\text{V}\cdot \text{s}$ [20,21]. From Figure 6, it has been proved that a REDC NP coated cell has a little bit of improvement in the generation rate curve. The difference between the maximum of the curves before and after the NP coating layer is calculated to be about 0.408%. That gives an indication that the conductivity impact of the coating nanoparticles has a major impact in the solar cell's efficiency increase rather than the optical conversions.

Electric field distribution before and after adding a REDC NP layer is shown in Figure 6a,b, respectively. There is some concentrated electric field between the solar cell and the REDC NP layer, with a slight difference in the electric field maximum value which was about 0.1340%. That could give a conclusion that the added layer of REDC NPs could have a slight optical impact in concentrating the electric fields inside the solar cells, in addition to the interface region between the cell and the coating layer. This confirms the mutual impact of the improved optical conversions and conductivity due to the REDC NP coating layer, with the dominant effect of the conductivity due to the hopping mechanism of the formed oxygen vacancies inside REDC NPs.

## 3. Experimental Section

### 3.1. Nanoparticles Synthesis

Reduced erbium-doped ceria nanoparticles have been synthesized using the chemical precipitation technique which is a relatively simple and inexpensive synthesis process [22]. Cerium (III) chloride (heptahydrate, 99.9%, Sigma-Aldrich Chemicals, St. Louis, MO, USA) of weight 0.485 g and erbium (III) chloride (heptahydrate, 99.9%, Sigma-Aldrich Chemicals, St. Louis, MO, USA) (0.015 g) are dissolved in de-ionized (DI) water (40 mL) to obtain a 3% weight ratio of erbium to cerium in the synthesized nanoparticles. This weight ratio is selected after a study by the authors of different weight ratios of erbium-doped ceria nanoparticles, synthesized using the same process, in which it was found that the optimal concentration of erbium in ceria for up-conversion is 3 wt % for the optimum improvement of solar cell efficiency. The solution is stirred constantly at 500 rpm in a water bath, while the temperature of the water bath is raised to 60 °C, and ammonia (1.6 mL) is then added to the solution. The solution is kept at 60 °C for 2 h and, then, the solution is stirred for another 22 h at room temperature. Then, the wet powder is dried, after being washed using ethanol, on a hot plate for 20 min. The thermal annealing of the dried nanoparticles is performed in a tube furnace (CM Furnace, Model 1730-20HT, Bloomfield, NJ, USA) with an atmosphere of hydrogen and nitrogen gases that are injected into the furnace at flow rates equal to 10 standard cubic feet per minute (scfm) for two hours at temperatures of 700 °C. The gases during the annealing assist with the reduction of the cerium ions from the Ce^4+^ to Ce^3+^ ionization states and the creation of the oxygen vacancies [23,24,25,26], while the thermal energy available during the high temperature anneal promotes the formation of the molecular energy levels of erbium inside the ceria host [15].

### 3.2. Characterizations of Nanoparticles

**Figure 8:**
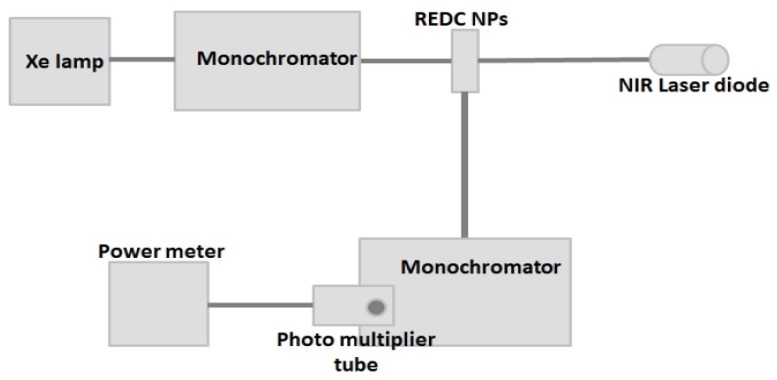
Up- and down-conversion fluorescence setup.

The optical absorption is measured using a dual-beam UV-Vis-NIR spectrometer (UV-3101PC Shimadzu, Tokyo, Japan). After the annealing procedure, a solution of nanoparticles is prepared with a concentration of 0.02 mg of nanoparticles in 10 mL of DI water. The colloidal solution is illuminated with both near-UV and near infra-red (NIR) excitations in an experimental apparatus that was designed to measure the down- and up-conversion process, as described in Figure 8. The fluorescence spectroscopy system consists of two excitation sources. The first one, the near UV excitation, is a Xenon lamp coupled to a monochromator, (Cornerstone 260, Newport, Irvine, CA, USA). The light that exits the monochromator (λ_exc_ = 430 nm) is focused on to the colloidal solution. The second one is an IR laser module of 780 nm. Both down- and up-conversion are detected using a second monochromator (Cornerstone 260, Newport, Irvine, CA, USA), positioned at a 90° angle to the first monochromator. The monochromator is scanned over the visible wavelength region and the fluorescence signal is detected by the photomultiplier tube (PMT 77340, Newport, Irvine, CA, USA), located at the exit port of the second monochromator. Then, the visible fluorescent emission is monitored using a power meter (2935C, Newport, Irvine, CA, USA).

Transmission electron microscope (TEM) (JEOL 1400, Peabody, MA, USA), is used to image the synthesized REDC NPs. The mean diameter of the nanoparticles is calculated using ImageJ software through Gaussian distribution of many size measurements. The operating parameters of the XRD (PANalytical X’Pert PRO, Amestrdam, The Netherlands), are 45 KV, 40 A and Cu Kα radiation (λ = 0.15406 nm). The conductivity of the solution of the synthesized nanoparticles is measured by A500 Orion meter (Thermo scientific, Tech Park, Singapore).

### 3.3. Coating Procedure

The synthesized nanoparticles are coated on the back sides of polycrystalline solar cells (2 inches × 2 inches) ordered from Solar Winds Inc., Austin, TX, USA. Coating has been operated using spin coater at 1500 rpm for a minute. Before coating, the electrodes on the backside of the cell have been covered through a scotch and released after the coating. That could avoid the direct contact between the nanoparticles and the metallic electrodes. The surface profile is detected using 3D optical surface profiler ZeGage (Zygo, Middlefield, CT, USA), with concentrating on the edge between coated cell and non-coated electrode to detect the thickness.

### 3.4. Solar Cell Characterization

The nanoparticle-coated and uncoated solar cells have been analyzed using a designed I–V characterization setup. The irradiance generated from a Xenon lamp (Oriel 100W, Irvine, CA, USA) followed by air mass (Newport AM1.5G, Irvine, CA, USA) is exposed to the coated/uncoated solar cells. Then, the different values of I and V are measured using Source meter 2400-C source meter (Keithley, Cleveland, OH, USA), with sweeping parameters as voltage range from −1 to +1 V through 1000 measuring points with 50 ms stoppage time per reading. Through the extracted *I*-values corresponding to the swept V-values, both I–V and P–V curves are drawn. From the I–V curve, some parameters could be measured such as filling factor, *V_o.c_*, *I_s.c_* and the optical efficiency. Using COMSOL Multiphysics software (COMSOL Inc., Burlington, MA, USA), generation rate and E-field distribution are analyzed with and without the nanoparticle layer on silicon solar cell.

## 4. Conclusions

This paper introduces a novel study of using reduced erbium-doped ceria nanoparticles (REDC NPs) as a coating layer on silicon solar cells. The presented work shows full optical characterization of the synthesized nanoparticles. The experimental results show the visible fluorescence emitted under both excitations of NIR and near UV. In addition, the results of the band gap and fluorescence confirm the formation of Ce^3+^ trap states which are associated with the formation of charged oxygen vacancies. That could increase the conductivity for any photo-generated electrons in the host NPs. When depositing REDC NPs on the back sides of solar cells, a promising improvement in the solar cell efficiency has been observed from 15% to 16.5% due to the mutual impact of improved electric conductivity and multi-optical conversions. In addition, the generation rate and maximum electric fields formed in the solar cells have been slightly improved due to the coating.
